# Functions of Forkhead Box O on Glucose Metabolism in Abalone *Haliotis discus hannai* and Its Responses to High Levels of Dietary Lipid

**DOI:** 10.3390/genes12020297

**Published:** 2021-02-20

**Authors:** Liu Wang, Yanlin Guo, Mingzhu Pan, Xinxin Li, Dong Huang, Yue Liu, Chenglong Wu, Wenbing Zhang, Kangsen Mai

**Affiliations:** 1 The Key Laboratory of Aquaculture Nutrition and Feeds (Ministry of Agriculture and Rural Affairs), the Key Laboratory of Mariculture (Ministry of Education), Ocean University of China, Qingdao 266003, China; wangliu@stu.ouc.edu.cn (L.W.); guoyanlin@stu.ouc.edu.cn (Y.G.); pmz@stu.ouc.edu.cn (M.P.); lixinxin0514@stu.ouc.edu.cn (X.L.); huangdong@stu.ouc.edu.cn (D.H.); liuyue@stu.ouc.edu.cn (Y.L.); kmai@ouc.edu.cn (K.M.); 2 School of Life Science, Huzhou University, 759 East 2nd Road, Huzhou 313000, China

**Keywords:** *Haliotis discus hannai*, forkhead box O, glucose metabolism, lipid, insulin sensitivity

## Abstract

The forkhead box O (FoxO) subfamily is a member of the forkhead transcription factor family. It has regulation functions in glucose metabolism in mammals and fish. In the present study, a gene of the *foxo* homolog in abalone *Haliotis discus hannai* was cloned. A conservative forkhead (FH) domain and a transactivation (FoxO-TAD) domain were identified. Abalone *foxo*-specific siRNA (small interfering RNA) was injected to investigate the functions of *foxo* on glucose metabolism. Knockdown of *foxo* inhibited expression of phosphoenolpyruvate carboxykinase (*pepck*) and significantly increased expressions of hexokinase (*hk*) and pyruvate kinase (*pk*), but it failed to inhibit the relative mRNA level of glucose-6-phosphatase (*g6pase*). Then, a 100-day feeding trial was conducted to investigate the response of *foxo* and glucose metabolism in abalone fed with 1.57% (LFD, low-fat diet), 3.82% (MFD, middle-fat diet) and 6.72% (HFD, high-fat diet) of dietary lipid, respectively. The insulin-signaling pathway (AKT) was depressed and FoxO was activated by the HFD, but it did not inhibit glycolysis (*hk*) or improved gluconeogenesis significantly (*pepck and g6pase*). At the same time, impaired hepatopancreas glycogen storage raised hemolymph glucose levels. In conclusion, abalone *foxo* can be regulated by dietary lipid and can regulate gluconeogenesis or glycolysis in response to changes of dietary lipid levels, in which glycogen metabolism plays an important role.

## 1. Introduction

Forkhead box O (FoxO) proteins are members of the O subfamily of the forkhead transcription factor family [1]. There is a “winged helix” or namely a “forkhead box” in their highly-conserved DNA-binding domain. In mammals, four FoxO genes (*foxo1*, *3*, *4*, and *6*) were found. FoxO proteins have multipurpose effects in animal systems, including functions related to cell survival, anti-oxidative stress, autophagy, and especially glucose metabolism [2,3]. Continuously-expressed FoxO1 proteins in transgenic mice can promote gluconeogenic gene expression in the liver, including phosphoenolpyruvate carboxykinase (*pepck*) and glucose-6-phosphatase (*g6pase*) [4], two crucial enzymes in gluconeogenesis [5]. FoxO6 plays a parallel role to FoxO1 in hepatic gluconeogenesis and one role compensates for functional loss of the other [6]. Depletion of FoxO6 attenuates this effect and protects against fat-induced glucose disorder in the liver [7]. On the other hand, FoxO proteins suppress the gene expression of glucokinase (*gk*) and pyruvate kinase (*pk*), and then inhibit glycolysis and glucose utilization [8,9].

In fish, *foxo1* in turbot was isolated and characterized. Knockdown of *foxo1* in primary hepatocytes inhibited the gene expression of *g6pase1* and *cpepck*, but the expression of *gk* was not significantly increased [10]. Previous studies in grass carp, *Ctenopharyngodon Idella,* [11] and hybrid grouper, *Epinephelus fuscoguttatus* ♀×*E. lanceolatus* ♂, [12] found that *foxo1* had effects on adipocyte differentiation and lipolysis. Genes of *foxo1*, *foxo3*, *foxo4,* and *foxo6* in channel catfish *Ictalurus punctatus* were also characterized. Furthermore, it was shown that these four *foxo* genes were significantly up-regulated in channel catfish after *Edwardsiella ictaluri* infection. It was suggested that *foxo* genes could play important roles in responses to bacterial infection [13].

As major targets of insulin action, transcriptional activities of FoxO proteins are suppressed by the insulin/PI3K/AKT signal pathway [14]. In response to insulin, FoxO1 is phosphorylated by activated AKT and then excluded to the cytoplasm, which results in it losing transcriptional activity [15]. FoxO proteins’ functions are regulated by nutrition intake. In fasting states, high levels of NAD^+^ and sirtuins are helpful in keeping FoxO proteins active to promote hepatic glucose production. Inversely, in fed states, the activities of FoxO proteins are inhibited in the mouse liver [16]. Excess dietary sugar inhibits the *Drosophila* homolog of FoxO transcription factors [15]. In high-fat-diet-fed db/db mice, transcriptional activity of *foxo1* was promoted compared to the normal-diet group and played a key role in mediating insulin resistance (IR) [17]. IR also appeared in the omnivorous Indian perch, *Anabas testudineus,* with long-term palmitate feeding [18]. But in rainbow trout, *Oncorhynchus mykiss,* hepatocytes cultured with higher concentrations of oleic acid, phosphorylation of FoxO1 was found to increase [19]. The above research indicates that the functions of FoxO on insulin-related glucose metabolism regulation largely depends on dietary nutrients, such as carbohydrates and lipids.

In aquatic invertebrates, *foxo* homologous have been identified in many species of mollusc, including the Pacific oyster, *Crassostrea gigas* [20], the owl limpet, *Lottia gigantea* [21], the Hong Kong oyster, *Crassostrea hongkongensis* [22], and the razor clam, *Sinonovacula constricta* [23]. Five single-nucleotide polymorphisms (SNPs) were identified in the coding region of *foxo* in the *S. constricta* [23]. All of the SNPs showed significant associations with total body weight, shell length, shell width, and shell height. Consequently, it was reported that *foxo* may be a gene related to growth traits and it may possess potential in the breeding-group selection of *S. constricta*. There is little research about the functions of *foxo* on glucose metabolism or other pathways of nutrition metabolism in molluscs.

Abalone, *Haliotis discus hannai,* is one of the precious species of mariculture molluscs in China. It is popular for its nutritional and medicinal value. Mai et al. [24] suggested that 3–5% of dietary lipid is optimal for better growth of abalone, and higher levels of dietary lipid resulted in excessive deposited lipid in tissues. In the study of Guo et al. [25], abalone fed with 3.82% of dietary lipid had the highest weight gain rate and increment in shell length compared with those fed with lower (1.57%, 2.34%, and 3.17%) or higher (4.63%, 5.56%, 6.17%, and 6.72%) dietary lipid level. Furthermore, the expression of genes involved in de-novo lipogenesis in the hepatopancreas was down-regulated by higher dietary lipid level (6.72%). However, there are no published studies on the regulation of glucose metabolism by FoxO in abalone, or whether high-fat diets affect its function. Therefore, the purpose of the present study was to explore the responses of abalone FoxO to high dietary lipid levels and its effects on the regulation of glucose metabolism. The results of this study will enrich theories about the role of FoxO in regulation of glucose metabolism in abalone. Meanwhile, it provides scientific instruction for the formulation of abalone feed related to dietary lipid levels.

## 2. Materials and Methods

### 2.1. Ethical Statement

All animal care and handling procedures in this study were approved by the Animal Care Committee of Ocean University of China.

### 2.2. Gene Cloning, Sequence Analysis, Tissue Distribution, and Function Analysis of foxo

#### 2.2.1. Animals and Sampling

Experimental abalones were obtained from a local seafood market in Qingdao, Shandong province, China. They were kept in the lucifugal seawater at 10–18 °C with continuous aeration, and fed with seaweed once daily for two weeks. After that, six abalone (body weight: 20.0 ± 0.1 g) were anesthetized by 5% of ethyl alcohol and used for isolation of tissues including the hepatopancreas, muscle, intestines, mantle, and gills. Samples were cleaned using saline solution and immediately frozen in liquid nitrogen, and then transferred to −80°C for the subsequent analysis.

#### 2.2.2. Cloning of *foxo* and Sequence Analysis

Total RNA in the hepatopancreas was extracted using Tissue Total RNA Isolation Kit (Vazyme, Nanjing, China). The purity and concentration of total RNA were measured with a Nanodrop 2000 (Thermo Fisher Scientific, USA) and the ratio of 260:280 was 1.8–2.0. The integrity of total RNA was tested by agarose gel electrophoresis. Complementary DNA (cDNA) was synthesized using PrimeScript^®^ RT reagent kit with gDNA Eraser (Takara, Japan).

The sequence of abalone *foxo* was searched from our transcript database of abalone (unpublished data). To amplify and confirm the complete CDS of abalone *foxo*, primers of CDS (*foxo*-F 5′ ATTACATCGCAGATTGGAG 3′ and *foxo*-R 5′ GACCGACAACCTCCCTGAT 3′) were designed using Primer Premier 5 and synthesized (Sangon Biotech, Qingdao, China). The PCR amplification was composed of 2 μL (200 ng/μL) of cDNA as template, 2 μL of each primer, 2 μL of 2× Taq Plus Master Mix (Vazyme, Nanjing, China), and ddH_2_O added to 50 μL. PCR was performed at 95 °C for 5 min then 35 cycles of 95 °C for 15 s, 59 °C for 15 s, 72 °C for 2 min, and 72 °C for 5 min. The products of PCR were separated by 1.2% agarose gel and the target band was purified using DNA Gel Extraction Kit (Beyotime, Shanghai, China). The purified DNA was then ligated into pEASY-T1 vector (TransGen, Beijing, China) for sequencing.

The cDNA sequence of *foxo* was aligned using the BlastX algorithm at the National Center for Biotechnology Information (http://www.ncbi.nlm.nih.gov/blast (accessed on 28 October 2020)) and similarity with other *foxo* gene sequences of different organisms was detected. The predicted protein sequence was deduced by the Expert Protein Analysis System (expasy) translate tool (https://web.expasy.org/translate/ (accessed on 28 October 2020)). The theoretical isoelectric point and molecular weight of FoxO protein were calculated by the expasy (https://web.expasy.org/cgi-bin/compute_pi/pi_tool(accessed on 28 October 2020)). The conserved domain in the amino acid sequence was predicted by the Simple Modular Architecture Research Tool (SMART: http://www.smart.emblheidelbergde/ (accessed on 28 October 2020)), and a phylogeny tree of neighbor-joining type was constructed using MAGE 7.0 software after multiple alignment of FoxO proteins using the ClustalW program in BioEdit software.

#### 2.2.3. Tissue Distribution

Tissues of six healthy abalones were isolated. The relative mRNA level of the *foxo* gene was investigated in the hepatopancreas, muscle, intestine, mantle, and gills by Quantitative real-time PCR (qPCR) using *β-actin* as reference gene based on the study of Cheng et al. [26].

#### 2.2.4. The *foxo* Interfering

Three abalone *foxo*-specific siRNAs (Table 1) were designed online (http://biodev.extra.cea.fr/DSIR/DSIR.html (accessed on 28 October 2020)). T7 RNAi Transcription Kit (Vazyme, China) was used to synthesize siRNAs. The scrambled siRNA (siRNA-NC) was also synthesized according to the sequences in a previous study [27]. The siRNAs (33 μg siRNA) were dissolved in RNase-free H_2_O (100 μL) and injected into the column muscle of abalone. There were six abalone in every group. In the control group, abalone were injected with 100 μL of RNase-free H_2_O. Then, 24 h after injection, the hepatopancreas of each abalone was collected to determine the interference efficiency by -qPCR. Afterwards, the group injected with the most-efficient siRNA was selected to detect the downstream genes expression.

#### 2.2.5. Quantitative Real-Time PCR (qPCR)

Total RNA was extracted from tissues, with the exception of the hepatopancreas, using TRIzol reagent (Invitrogen, USA) and the Tissue Total RNA Isolation Kit (Vazyme, Nanjing, China). The quality of total RNA was tested by the same procedure as mentioned above in Section 2.2.2. Then, complementary DNA (cDNA) was synthesized using HiScript^®^ II Q RT SuperMix for qPCR (+gDNA wiper) (Vazyme, Nanjing, China) and then diluted three times with DEPC-treated water. The resulting products were used as template for amplification. qPCR was performed in a quantitative thermal cycle (Mastercycler^®^ eprealplex; Eppendorf, Germany).

Gene expression of *foxo* was detected to determine its tissue distribution in abalone. In the *foxo*-interfering test, target genes included *foxo*, hexokinase (*hk*), *pk*, *pepck*, and *g6pase*. The reaction was in a total volume of 15 μL (7.5 μL of ChamQTM Universal SYBR^®^ qPCR Master Mix (Vazyme, Nanjing, China), 0.3 μL (10μM) of each primer, 1 μL of cDNA, and 5.9 μL of DEPC-treated water) and underwent the following process: 95 °C for 2 min, followed by 40 cycles of 95 °C for 10 s, 58 °C for 10 s, and 72 °C for 20 s. Gene expression levels were rectified to *β-actin* mRNA levels according to the previous study [25] and relative gene expression was quantified with the 2^−ΔΔ^ method. All the primers used in this study were previously tested for amplification efficiency. The sequences of all primers used in the present study are shown in Table 2.

### 2.3. Feeding Trial

#### 2.3.1. Experimental Diets

The experimental diets were prepared using fish meal and soy protein concentration as the main protein sources, fish oil and soybean oil as the main lipid sources. Three isonitrogenous (30% protein) experimental diets were formulated to contain 1.57% (LFD, low-fat diet), 3.82% (MFD, middle-fat diet), and 6.72% (HFD, high-fat diet) of lipid, respectively (Supplementary Materials Table S1).

#### 2.3.2. Feeding and Sampling

A 100-day feeding trial was performed in the natural sea area of Pingtan, Fujian Province, China. Abalone were obtained from a local commercial farm and acclimatized to the experimental environment for 14 days. Then a completely random design was adopted to assign abalone (body weight: 10.98 ± 0.05 g) into three groups and fed with the three experimental diets, respectively. There were three replicates per group, and 60 abalones per replicate (sea cage). Abalones were fed once every 2 days. The feces and uneaten diets were removed to maintain water quality. During the feeding trial, water temperature ranged from 15 to 28 °C, pH 7.9–8.3, and the dissolved oxygen was not less than 6 mg/L. At the end of the feeding trial, the hemolymph and hepatopancreas of abalone were sampled after being fasted for three days and anesthetized by 5% ethyl alcohol. The hemolymph samples were collected and centrifuged (3000× *g*, 10 min, 4 °C). And then the plasma was collected and stored in −20 °C. Abalone hepatopancreas were cleaned using saline solution and immediately frozen in liquid nitrogen, and then transferred to −80 °C for subsequent analysis.

#### 2.3.3. Biochemical Parameters

The glucose, insulin, leptin, and adiponectin contents in hemolymph were analyzed. The glycogen content and activities of PK, HK, PEPCK, and glucose6-phosphate dehydrogenase (G6PDH) in the hepatopancreas were analyzed. The insulin, leptin, and adiponectin contents were determined using a double-antibody sandwich enzyme-linked immunosorbent assay (ELISA) (shellfish-specific). The kit for leptin (YX-120516S) was purchased from Sino Best Bio, Shanghai, China. Kits for insulin (ml601411) and adiponectin (ml208360) were purchased from MLbio, Shanghai, China. In the ELISA procedure, the standard 50 μL with different concentrations was added to the standard wells to make a standard curve. Then 10 μL of hemolymph sample and 40 μL of diluent were added into testing wells. The blank well was empty. Then, horseradish peroxidase (HRP)-labeled antibody (100 μL) was added into each standard well and sample well. The ELISA plate was incubated and thoroughly washed. The substrate TMB was used to develop color and the absorbance at 450 nm was determined to calculate hormone concentration. The content of hemolymph glucose (A154-1-1) and glycogen (A043-1-1), and activities of PK (A076-1-1), HK (A077-3-1), and PEPCK (A131-1-1) in the hepatopancreas were determined using commercial kits from Nanjing Jiancheng Bioengineering Institute, Nanjing, China. The kit for activity of G6PDH (BC0265) was from Solarbio, Beijing, China. For glucose determination, 10 μL of hemolymph sample was oxidized and colored by glucose oxidase and peroxidase. Then absorbance at 505 nm was determined. To analyze glycogen, 50 mg of hepatopancreas samples was put into the test tube and 150 μL of hydrolysate was added. Then the tubes were put into a boiling water bath for 20min and then diluted to 1%. Anthrone colorimetry was used for color development and concentration determination. For PK and HK determination, 0.05 g of hepatopancreas tissue was weighed and 450 μL of normal saline was added. The tissue was homogenized on ice and centrifuged (7000× *g*, 10 min) for 1% supernatant. Then 20 and 30 μL of supernatant was taken, respectively, for PK and HK activity analysis. In the PEPCK and G6PDH determination, 0.1 g of hepatopancreas tissue and 1 mL of extracting solution were, respectively, added into a tube to homogenize and centrifuged as mentioned above. Then 50 μL and 10 μL of supernatant was taken, respectively, for activity analysis.

#### 2.3.4. Quantitative Real-Time PCR (qPCR)

After the feeding trial, gene expression of *foxo* in the abalone hepatopancreas was detected. Other target genes included *pepck*, *g6pase*, *hk*, *pk*, *tk*, *g6pdh*, *gsk3β*, *glut1*, *pyg* and *pgc1α*. The method was the same as that in Section 2.2.5.

#### 2.3.5. Western Blot Analysis

Tissues of hepatopancreas were homogenized in RIPA buffer (high) (Solarbio, Beijing, China) with protease and phosphatase inhibitor cocktails (Roche, Switzerland) on ice. After 10 min, the homogenate was cleared by centrifugation at 12,000× *g* for 30 min. Protein concentrations were determined with a BCA Protein Quantification Kit (Vazyme, Nanjing, China), and then protein samples were diluted to 1.5 μg/μL with RIPA buffer (high). Protein samples (15 μL protein per lane) were separated by SDS-PAGE and transferred to 0.45 μm PVDF membrane (Millipore, USA) for Western blot analysis. The membrane was blocked with 5% nonfat milk in TBST buffer (20 mM Tris·HCl, 500 mM NaCl, 0.1% Tween 20) for 1 h at room temperature. After being washed by TBST three times, the membrane was incubated with primary antibody overnight at 60r/min, 4 °C before horseradish peroxidase (HRP)-conjugated secondary antibodies were added and incubated for 1h at room temperature. The membrane was visualized using ECL reagents (Vazyme, Nanjing, China). The primary antibodies are as follows: antibodies against reduced glyceraldehyde-phosphate dehydrogenase (GAPDH) (Cell Signaling Technology Inc., #2118), FoxO (Cell Signaling Technology Inc., #9472), protein kinase B (AKT) (Proteintech, 60203-2-Ig), phospho-AKT (Thr308) (Affinity Biosciences Cat#AF3262),) and phospho-FoxO (Ser319) (Wanleibio, WL03634). The Western bands were quantified using ImageJ software.

### 2.4. Statistical Analysis

Data are expressed as the mean ± S.E and were analyzed in SPSS 25.0. For efficiency of siRNA interference, the *T*-test was used to compare with the control group and one-way analysis of variance (ANOVA) was used for the tissue distribution of *foxo* and the effects of different dietary lipid levels. Significant differences among groups were examined by Tukey’s multiple range test (95% confidence interval).

## 3. Results

### 3.1. Identification of foxo in Abalone

**Figure 1:**
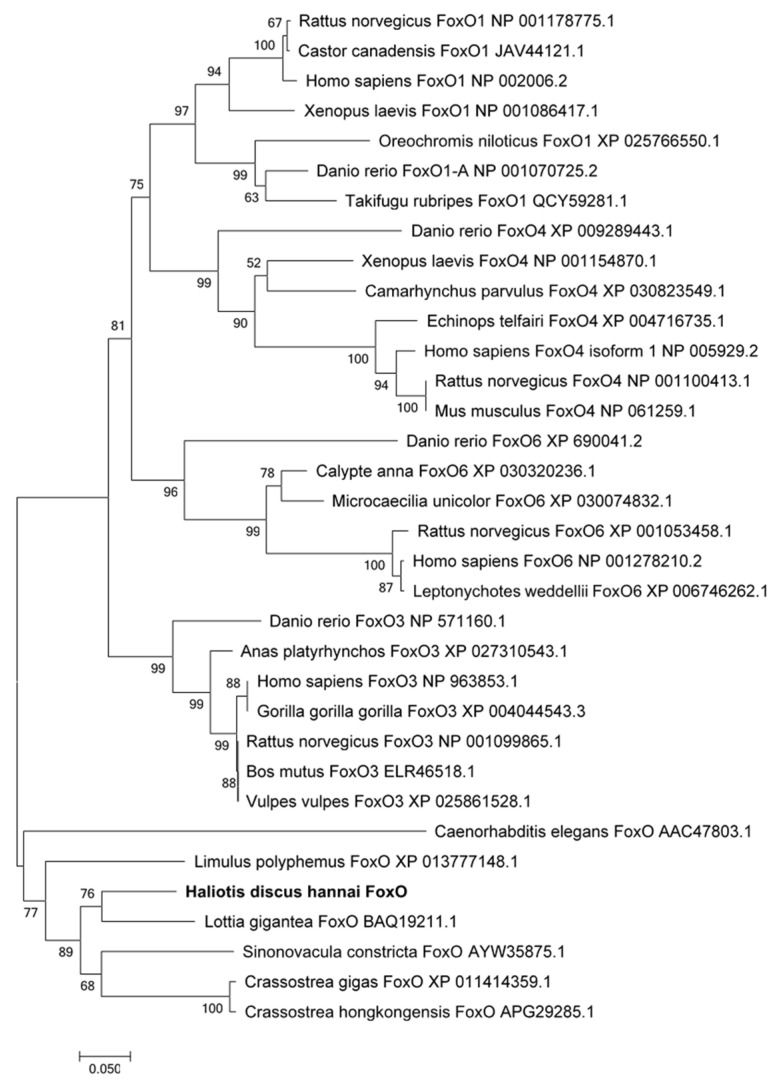
Phylogenetic tree based on the sequences of forkhead (FH) domains of forkhead box Os (FoxOs) from abalone and other species.

**Figure 2:**
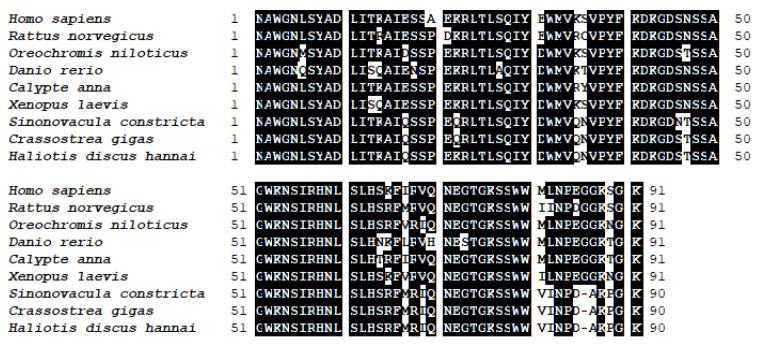
Multiple alignment of amino acid sequence of FH domain from abalone and other organisms. *Crassostrea gigas* (XP_011414359.1), *Sinonovacula constricta* (AYW35875.1), *Xenopus laevis* (NP_001086417.1), *Calypte anna* (XP_030320236.1), *Danio rerio* (XP_009289443.1), *Oreochromis niloticus* (XP_025766550.1), *Rattus norvegicus* (NP_001099865.1), and *Homo sapiens* (NP_002006.2). Identical residues are shaded black.

The *foxo* gene was cloned from abalone and was predicted to encode 641 amino acids (aa) (GenBank Accession No: MN864138). The molecular weight of speculative protein was 69.84 KDa, and the theoretical isoelectric point was 5.23. Abalone FoxO contains a conservative forkhead (FH) domain (64–153aa) and a transactivation (FoxO-TAD) domain (590–622 aa) (Figure S1). The result of BLAST analysis indicated that the deduced amino acid sequence of abalone FoxO shares higher identities with other reported invertebrate FoxO. It has 55% homology with *Lottia gigantea* (BAQ19211.1) and 47% with *Crassostrea gigas* (XP_011414359.1). The phylogenetic tree (Figure 1) was constructed with the FoxOs of various species. It shows that abalone FoxO is in a branch with other invertebrate FoxO. This branch has a distant relationship with vertebrate FoxO6, FoxO4, FoxO3, and FoxO1. Multiple alignments revealed that the FH domain of abalone FoxO is consistent with FoxOs from other species (Figure 2).

### 3.2. Tissue Distribution of foxo in Abalone

**Figure 3:**
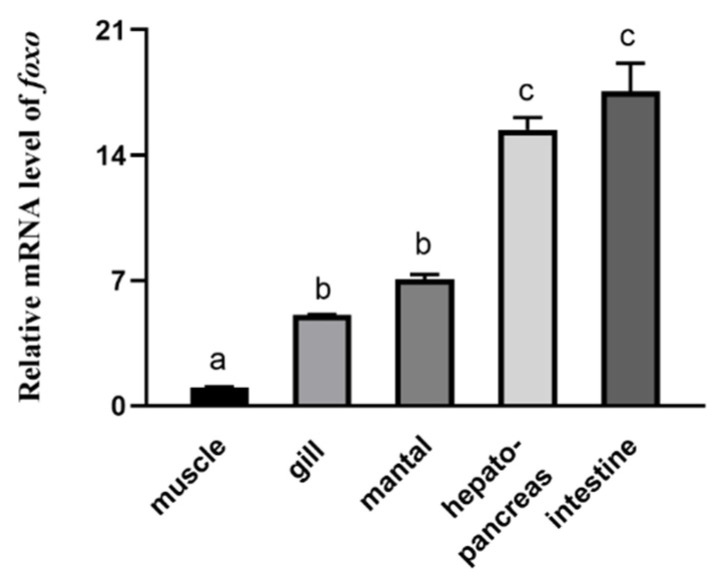
Tissues distribution of FoxO in abalone. Data are presented as means ± S.E., *n* = 6. Values with different letters are significantly different (*p* < 0.05).

The mRNA of abalone *foxo* was detected in the hepatopancreas, muscle, intestines, mantle, and gills (Figure 3). qPCR showed that abalone *foxo* has the highest expression in the intestine and hepatopancreas, followed by the mantle and gills. The expression of *foxo* is lowest in muscle.

### 3.3. Knockdown of foxo In Vivo in Abalone

**Figure 4:**
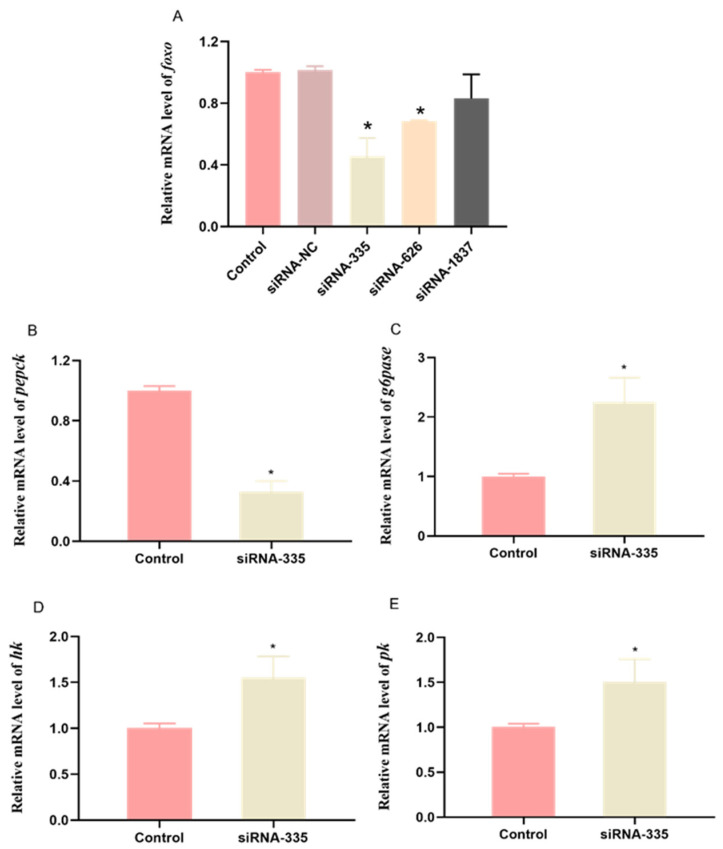
Relative expressions of *foxo* and glucose-related genes in the hepatopancreas of abalone at 24 h after *foxo*-specific siRNA injection. The mRNA levels of forkhead box O *(foxo*) (**A**), phosphoenolpyruvate carboxykinase (*pepck*) (**B**), glucose-6-phosphatase (*g6pase*) (**C**), hexokinase (*hk*) (**D**), and pyruvate kinase (*pk*) (**E**) were evaluated using qPCR. Expression values were normalized with expressions of *β-actin*. Values are represented as mean ± S.E., *n* = 6. * represents significant difference between control and siRNA-335 groups (*p* < 0.05).

To determine the interfering efficiency of three targeting siRNAs and scramble siRNA, -qPCR was performed to detect the relative mRNA level of *foxo*. The results are shown in Figure 4A. Compared with control group, the scrambled siRNA (siRNA-NC) did not affect the gene expression level of *foxo*, and siRNA-335 was the most efficient at decreasing the mRNA level of *foxo* in the hepatopancreas.

At the 24th hour after injection of siRNA-335, the mRNA levels of phosphoenolpyruvate carboxykinase (*pepck*) significantly decreased, while mRNA levels of glucose-6-phosphatase (*g6pase*) was significantly higher than that in the control group (*p* < 0.05) (Figure 4B,C). Both hexokinase (*hk*) (Figure 4D) and pyruvate kinase (*pk*) (Figure 4E) had significantly higher transcriptional levels than that in the control group (*p* < 0.05).

### 3.4. Hemolymph and Hepatopancreas Parameters after Feeding Trial

**Figure 5:**
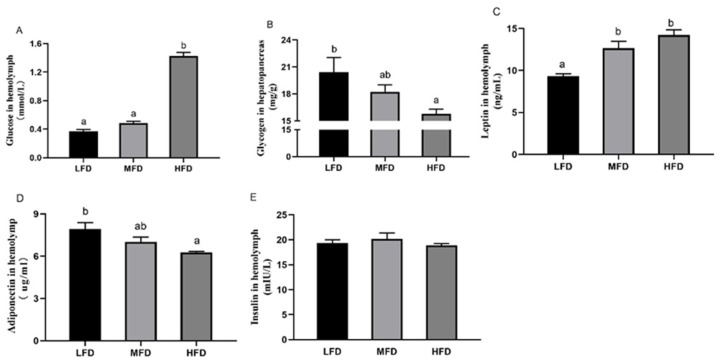
Hemolymph and hepatopancreas parameters after feeding trial. The contents of glucose in hemolymph (**A**), glycogen in hepatopancreas (**B**) and leptin (**C**), adiponectin (**D**) and insulin (**E**) in hemolymph were measured. LFD, low-fat diet; MFD, middle-fat diet; HFD, high-fat diet. Data are presented as mean ± S.E., *n* = 3. Values with different letters are significantly different (*p* < 0.05).

**Figure 6:**
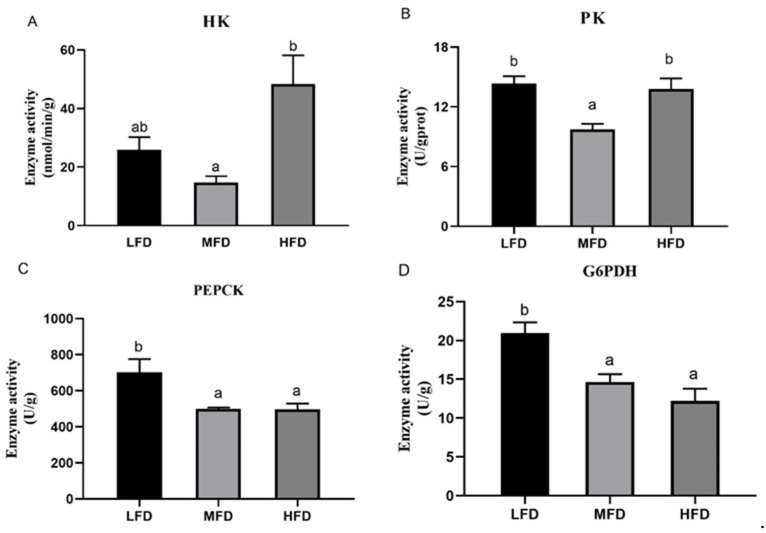
Enzyme activities in the hepatopancreas of abalone after feeding trial. Activities of, hexokinase (HK) (**A**), pyruvate kinase (PK) (**B**), phosphoenolpyruvate carboxykinase (PEPCK) (**C**) and glucose6-phosphate dehydrogenase (G6PDH) (**D**) were measured. Values are represented as mean ± S.E., *n* = 3. Values with different letters are significantly different (*p* < 0.05).

The data are shown in Figure 5 and Figure 6. In hemolymph, the content of glucose (Figure 5A) was significantly higher in the HFD group (*p* < 0.05), while there was no significant difference between the other two groups (*p* > 0.05). The content of glycogen in the hepatopancreas (Figure 5B) declined gradually with the increasing dietary lipid levels (*p* < 0.05). The contents of leptin (Figure 5C) in the HFD and MFD groups were higher than that in the LFD group (*p* < 0.05). The level of adiponectin in hemolymph (Figure 5D) was highest in the LFD group and lowest in the HFD group (*p* < 0.05). There was no significant difference in the contents of insulin (Figure 5E) among the three groups (*p* > 0.05).

Activity of HK had the highest value in the HFD group and the lowest value in the MFD group (*p* < 0.05) (Figure 6A). Activity of PK decreased in the MFD group and increased again in the HFD group (*p* < 0.05) (Figure 6B). The MFD and the HFD groups showed lower PEPCK activities than the LFD group (*p* < 0.05) (Figure 6C). Activity of G6PDH in the LFD group was significantly higher than those in the MFD and the HFD groups (*p* < 0.05), but there was no significant difference between the MFD and the HFD groups (*p* > 0.05) (Figure 6D).

### 3.5. Effects of Dietary Lipid on Gene and Protein Expressions in Hepatopancreas

**Figure 7:**
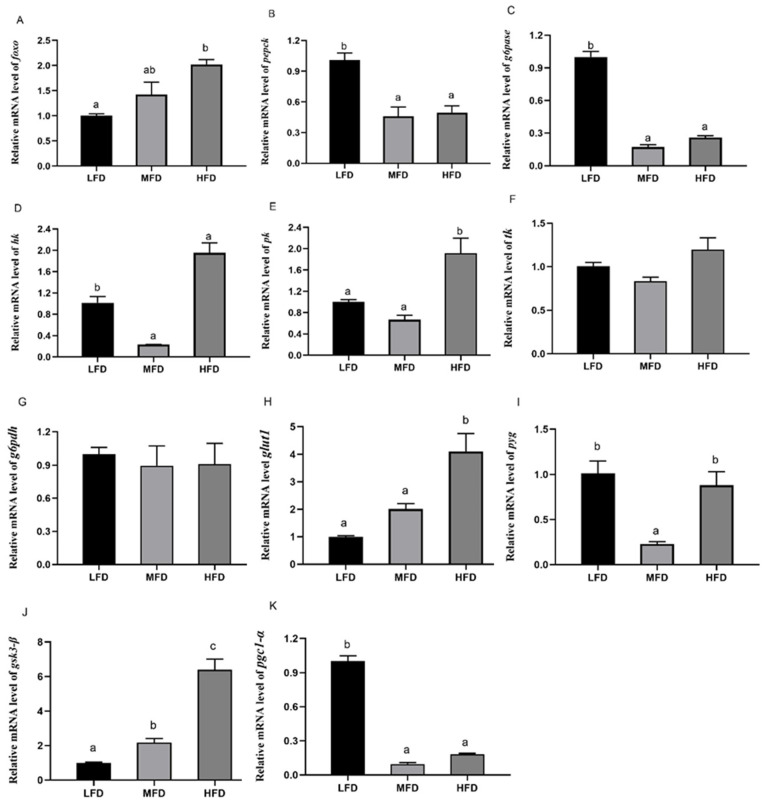
Relative mRNA levels in the hepatopancreas of abalone after feeding trial. The mRNA levels of *foxo* (**A**), phosphoenolpyruvate carboxykinase (*pepck*) (**B**), glucose-6-phosphatase (*g6pase*) (**C**), hexokinase (*hk*) (**D**), pyruvate kinase (*pk*) (**E**), transketolase (*tk*) (**F**), glucose-6-phosphate dehydrogenase (*g6pdh*) (**G**), glucose transporter 1 (*glut1*) (**H**), glycogen phosphorylase (*pyg*) (**I**), glycogen synthase kinase-3β (*gsk3-β*) (**J**), and peroxisomal promoter receptor co-activator 1α (*pgc1-α*) (**K**) were evaluated using qPCR. Values are represented as mean ± S.E., *n* = 3. Values with different letters are significantly different (*p* < 0.05).

**Figure 8:**
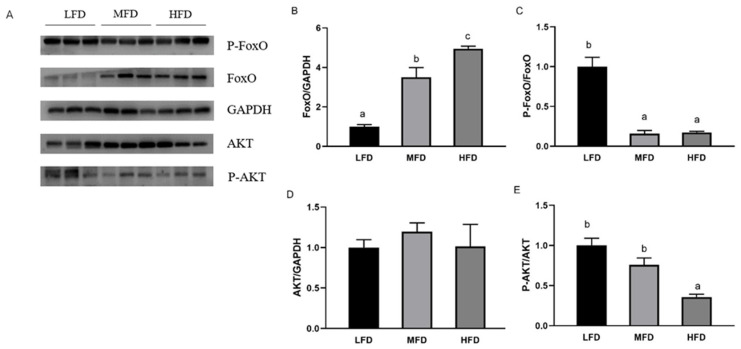
Protein levels in the hepatopancreas of abalone after feeding trial (**A**). The relative protein abundances of FoxO (**B**), P-FoxO (**C**), AKT (**D**), and P-AKT (**E**) in hepatopancreas were measured by Western blot and expressed as relative expression values to those in the LFD group. Values are represented as mean ± S.E., *n* = 3. Values with different letters are significantly different (*p* < 0.05).

The relative mRNA levels of *foxo* (Figure 7A) in the MFD and the HFD groups significantly increased with the increase in dietary lipid levels. Compared to that in the MFD group, the mRNA levels of *pepck* (Figure 7B) and *g6pase* (Figure 7C) increased significantly in the LFD group (*p* < 0.05) and slightly increased in the HFD group (*p* > 0.05). The transcriptional levels of *hk* (Figure 7D) and *pk* (Figure 7E) were significantly higher in the HFD group (*p* < 0.05) and *hk* had the lowest transcriptional levels in the MFD group (*p* < 0.05). No significant difference was present on gene transcriptional levels of *tk* (Figure 7F) and *g6pdh* (Figure 7G) among all of groups (*p* > 0.05). The gene expression of *glut1* (Figure 7H) increased with increasing lipid level and was highest in the HFD group with a significant difference (*p* < 0.05). In the MFD group, the relative mRNA level of *pyg* (Figure 7I) was lowest (*p* < 0.05) and gene expression of *gsk-3β* (Figure 7J) elevated gradually with significant differences in each group (*p* < 0.05). The transcriptional level of *pgc1-α* (Figure 7K) showed no difference between the MFD and the HFD groups and was highest in the LFD group (*p* < 0.05). At the same time, protein levels were detected (Figure 8A). The protein level of FoxO (Figure 8B) increased as lipid levels increased (*p* < 0.05). The LFD group showed the highest level of phosphorylation of FoxO (*p*-FoxO) (Figure 8C) (*p* < 0.05). The protein level of AKT increased in the MFD group and then decreased in the HFD group, but there was no significant difference (Figure 8D). Phosphorylation of AKT (P-AKT) level (Figure 8E) in the HFD group was significantly lower than that in the other two groups (*p* < 0.05).

## 4. Discussion

FoxO proteins were firstly discovered in mammals. The orthologs of mammalian FoxOs were also characterized in invertebrates such as *Drosophila melanogaster* [28], *Caenorhabditis elegans* [29], *Blattella germanica* [30], and some shellfish [20,21,22,23]. In the present study, the *foxo* in abalone was cloned and the full-length ORF of *foxo* was 1926 bp, encoding peptides of 641 amino acids. Phylogenetic analysis showed that the abalone FoxO shares a higher degree of similarity with FoxO orthologs of molluscs and elegans than other FoxO proteins from vertebrates including the human, mouse, and some fish. Tissue distribution revealed that gene expression of *foxo* was high in the intestine. In *Drosophila*, it has been confirmed that intestinal FoxO signaling is necessary to survive oral infection [31]. After *E. ictalurid* infection, four *foxo* genes were significantly up-regulated in the intestine of channel catfish [13]. Therefore, it was indicated that FoxO plays a part in the intestinal immune system of abalone. In addition, the higher expression of *foxo* in the gills and mantle suggests its potential function in responses to the oxidative stress [32]. High gene expression of *foxo* in the hepatopancreas was consistent with that in turbot [10], grass carp [11], and the mouse [33]. In mammals, the liver is the key organ participating in nutrient metabolism to maintain blood glucose level [34], so, the present study paid attention to the effects of FoxO on the glucose metabolism in abalone.

In mice, FoxO proteins have a synergistic effect on liver glucose production. Compared with knocking out FoxO1 alone, knocking out FoxO1/3/4 enhances higher glucose tolerance and insulin sensitivity [35,36]. FoxO proteins can bind to the promoters of *pepck* and *g6pase* to active gene transcription, thereby promoting the process of gluconeogenesis [37]. Previous studies showed that FoxO proteins suppress the expression of glucokinase, which contains several mechanisms, such as hepatocyte nuclear factor-4 (HNF-4) and peroxisome proliferator receptor γ (PPARγ) [4,16]. FoxO proteins interact with HNF-4 and PPARγ and inhibit their contribution to the expression of glucokinase [38,39]. In the present study, expression of *pepck* was inhibited, and expressions of *hk* and *pk* in the hepatopancreas significantly increased after the knockdown of *foxo* in abalone. These results were similar to those in mammals. However, the siRNA-335 treatment failed to inhibit the expression of *g6pase*. In the previous study, the deduced amino acid sequence of abalone *g6pase* shares high identity with California sea hare *g6pase2* (48%) and Pacific oyster *g6pase2* (42%) [40]. And in turbot, *g6pase2* was also increased significantly after knockdown of *foxo1* in primary hepatocytes [10]. This suggested that inhibition of *foxo* resulted in increase in glycolytic gene expression but failed to reduce gluconeogenesis in the hepatopancreas of abalone.

Carbohydrates and lipids are two important energy sources in body and are related closely to the regulation of metabolic homeostasis. A high-carbohydrate diet might lead to a high level of plasma insulin, and further deposition of glycogen and lipids in liver and muscle. Meanwhile, it results in the dysregulation of glucose homeostasis [41,42]. However, carbohydrate metabolism is not the sole cause of dysglycemia; lipid metabolism is also one of the reasons [43,44]. Previous studies showed that high dietary fat could result in insulin resistance (IR) as well as increased lipolysis and fatty acid oxidation [45,46,47]. IR is defined as impaired insulin action resulting in insulin insensitivity in target organs including the liver, skeletal muscle, and adipose tissue [48]. Despite many years of research, the exact mechanism of IR remains unclear. In mammals, constitutive FoxO activation in the liver and pancreatic β cells causes hyperglycemia and mediates IR [49,50]. Reduction of FoxO1 mediating by antisense oligonucleotide improved glucose tolerance and peripheral IR in liver or adipose tissue of diet-induced obese mice [51,52]. Based on the above data, a feeding trial was performed, in the present study, to ascertain the effects of *foxo* on glucose metabolism in abalone under the stress of high dietary lipid levels.

In the HFD group of the present study, the hemolymph glucose level in abalone increased, which was consistent with levels in the blunt snout bream, *Megalobrama amblycephala* [53], tilapia, *Oreochromis niloticus* [54], and rainbow trout, *Oncorhynchus mykiss* [55]. The content of hepatopancreatic glycogen decreased gradually with the increase of dietary lipid content. The most obvious pathological feature of liver IR is that the dysfunction of gluconeogenesis and glycogenolysis leads to increased glycogen output from liver [56,57]. Adiponectin was reported helpful to improve insulin sensitivity and correct disturbances in whole-body glucose homeostasis induced by a high-fat diet, but leptin increases insulin resistance under a high-fat diet [58,59,60,61,62,63]. In the present study, the increase in leptin and decrease in adiponectin suggest that an HFD reduced the insulin sensitivity of abalone. The same results appeared in C57BL/6J mice fed with a high-fat diet [64,65,66]. In contrast, in the LFD group of the present study, adiponectin increased and leptin decreased compared with those in the MFD group, but there was no difference in glucose levels between the LFD group and the MFD group. Subsequently, the responses of FoxO in the hepatopancreas of abalone fed with different dietary lipid levels were determined. Also, downstream gene-expression-related glucose synthesis and utilization were analyzed to elucidate the underlying mechanism.

Compared with the MFD group, down-regulation of FoxO protein expression level and up-regulation of phosphorylation of FoxO indicated that the transcriptional activity of FoxO was inhibited in the LFD group. In the HFD group, however, protein expression of FoxO was significantly increased, and the two glycolytic enzymes (HK and PK) had higher gene expressions and activities than those in the MFD group. Similarly, a high-fat diet promoted gene expression of glucokinase (*gk*) in blunt snout bream *Megalobrama amblycephala* with a high level of blood glucose [53]. Because glycolysis is an important outlet pathway of blood glucose, the hypothesis might be that enhanced PK and HK (the isoenzyme of glucokinase) with high glucose level are a physiological adaption to the hyperglycemia [54,67]. In the LFD group, expressions of *hk* and *pk* were elevated compared to the MFD group. Meanwhile, the protein level of FoxO was lowest and its phosphorylation level increased. As the dietary lipid content decreased, in the present study, activities of G6PDH in the LFD group increased. G6PDH is a rate-limiting enzyme in the pentose phosphate pathway that produces NADPH for biosynthesis of fatty acids and cholesterol [68,69]. Presumably, due to the lack of dietary lipid in the LFD group, more glucose is converted into lipids in the body [70]. With respect to gluconeogenesis, compared to the LFD group, gene expression or activities of two key enzymes (PEPCK and G6pase) reduced in the MFD and the HFD groups that were fed more lipid, but the protein levels of FoxO of the two groups increased and the phosphorylation levels of FoxO decreased significantly. These results suggest that higher lipid content in diets could have elevated the activity of FoxO, but did not promote gluconeogenesis. This seems to be contradictory with previous studies in mammals that found enhanced gluconeogenesis induced by high-fat diet or in diabetes [56,71]. Moreover, rainbow trout fed a high-fat diet had postprandial hyperglycemia and increased G6Pase activity over the two weeks [55]. In the present study, the mechanism of different variation trends between FoxO and gluconeogenesis (*pepck* and *g6pase*) in abalone was not clear. Previous studies showed that FoxO also interacts with glucocorticoids, PGC1-α, and some factors in gluconeogenesis [16,72,73,74]. PGC1-α is transcriptional coactivator with FoxO and regulated by glucocorticoids to raise the level of blood glucose by regulating gluconeogenesis [74,75,76]. In PGC-1-deficient mice, mRNAs encoding PEPCK and G6Pase reduced, along with fasting glucose levels [77]. The PGC-1 family plays an active role in mitochondrial metabolism whose dysfunction leads to decreased fatty acid β-oxidation and ectopic accumulation of fat [78,79]. In mice under oxidative stress induced by high dietary fat, it has been found that protein level of PGC1-α was suppressed [80]. Although FoxO was activated by an HFD, in the present study, the gene expression of PGC1-α was not elevated significantly, which is consistent with the expressions of *pepck* and *g6pase*. However, in mammals, there is a paradox in the regulation of PGC-1 by high-fat diet. Gu et al. [81] reported that gene expression of *pgc1-α* was significantly elevated in rats fed with a high-fat diet. In the meanwhile, activity of FoxO1 and relative mRNA levels of *pepck* and *g6pase* were higher than those in the control group. These results indicated that the regulation mechanism of HFD on the FoxO/PGC1-α pathway and gluconeogenesis in abalone are not clear and remain to be discovered.

Previous studies have found that adipocytokines (e.g., resistin, tumor necrosis factor-α, retinol binding protein 4, and chemerin) expressed and secreted by adipose tissue interfere with the phosphorylation of insulin receptor or insulin-receptor substrate [82,83,84]. When phosphorylation of insulin receptor substrate is impaired, phosphorylation of AKT and the effective transmission of insulin signals will be affected [85,86]. To investigate the efficiency of the insulin pathway, protein levels of AKT and phosphorylation of AKT were measured in the present study. Compared with those in the other two groups, down-regulation of phosphorylation of AKT was found in the HFD group. Similar results were also showed in db/db and HFD mice [17,80]. Besides FoxO, GSK3-β is also a key inhibition target of P-AKT and it then inhibits the synthesis of glycogen through phosphorylating glycogen synthase [87]. Overexpression or abnormal activation of GSK3-β was associated with type 2 diabetes mellitus [88,89]. In the HFD group, in the present study, high expression levels of *pyg* and *gsk3-β* indicated an increase in hepatic glycogen decomposition and a decrease in its synthesis, along with decreased glycogen content. Low levels of liver glycogen, induced by high-fat diet, were also found in diabetic mice [90]. Liver glycogen is one of the main forms of carbohydrate storage in the body and the content of liver glycogen is very important in stabilizing blood glucose levels [91]. Impaired glucose storage and decreased glycogen content rather than gluconeogenesis might be one of the reasons of hyperglycemia in abalone in the HFD group.

In conclusion, the activity of FoxO and hemolymph glucose level in abalone were elevated by high dietary lipid levels. Meanwhile, gene expression and activities of the two key enzymes (PEPCK and G6Pase) in gluconeogenesis did not increase, but under high lipid levels, impaired hepatopancreas glycogen storage (increased *gsk3-β*) and content raised the glucose level in hemolymph. Therefore, insulin signaling (AKT) bifurcates at FoxO and GSK3-β to regulate gluconeogenesis and glycogen metabolism in abalone. Underlying mechanisms of the synergistic effect of FoxO and other transcription factors as well as their effects on glucose metabolism under different nutrition status require further study.

## Figures and Tables

**Table 1:** siRNAs used in the RNA interference assay.

Name	Target Sequence
siRNA-335	CAGCTGGTTGGAAGAATTC
siRNA-626	CACGAGCTAGTTCTAATGC
siRNA-1837	AAGCAAGAGTTGTCTCTAG
siRNA-NC	TTCTCCGAACGTGTCACGT

**Table 2:** List of the primers used for the real-time PCR analysis.

Genes	5′/3′ Forward Primer	5′/3′ Reverse Primer	Accession Number
*foxo*	AATGGCCCTGTTTCAACCAC	CTTCCCGACTGTAAAGGTGT	MN864138
*pepck*	TCGACAACAATGGCAAGCTC	CTTGTCTCCGCAACATTCGT	MH220521.1
*g6pase*	CGTACAAACTGCCTCACTCG	ATTCTCGGGACAATGTTCACAA	LC456704.1
*hk*	ACGCCAGATCAACTCTCGAA	GCATCACACGCTTGTAGGTCA	MH220519.1
*pk*	AGGCAAGAACATCCGCATC	CTCCTGCAAGATCTCATCGAAC	MH220522.1
*tk*	TCCCAGAACGCTTCATCGAG	CACAGCCAATTCCGACACCA	MT887625
*g6pdh*	TGCCACCATCAGTCTTCGAG	AGATCATGTCCAAACGGCTT	MT551204
*gsk3β*	AACTGTTCCGAAGTCTTGCAT	ATATCCCGATGACAAACTCCT	FJ435173.1
*glut1*	TCCAGTTTGGCTACAATACAGG	CCCATTCCGATCAAAGTAGGTC	MT551207
*pyg*	TCTCGTGTTCTGTACCCCAA	TGAACCTGCGTACAATGTCC	LC456706.1
*pgc1α*	CGAAGACCCAGCAGTCACC	TAACGATCAGTGTCACGACCT	MT873877
*β-actin*	CCTCAAGTACCCCATCGAGCAC	ATCTTCTCCATGTCGTCCCAG	AY380809.1

## Data Availability

The data that support the findings of this study are available from the corresponding author upon reasonable request.

## Supplementary Materials

The following are available online at https://www.mdpi.com/2073-4425/12/2/297/s1, Figure S1: Nucleotide and deduced amino acid sequences of FoxO in abalone. The forkhead (FH) domain (64–153aa) is marked in yellow and a transactivation (FoxO-TAD) domain (590–622aa) is underlined. Table S1: Formulation and proximate composition of the experimental diets.
