# Supplementary material for: Functions of Forkhead Box O on Glucose Metabolism in Abalone Haliotis discus hannai and Its Responses to High Levels of Dietary Lipid
Source: Genes (Basel). 2021 Feb 20;12(2):297. doi: 10.3390/genes12020297 (PMC7924355; doi:10.3390/genes12020297)
Supplement: Supplementary file 1 [file genes-12-00297-s001.pdf]

Table S1. Formulation and proximate composition of the experimental diets.

Ingredients	Diets		
	LFD	MFD	HFD
Fish meal	1.00	1.00	1.00
Soy protein concentrate	6.50	6.50	6.50
Wheat gluten	5.00	5.00	5.00
Dehulled soybean meal	23.40	24.25	25.38
Strong flour	29.30	26.32	22.34
Fish oil and soybean oil (1:1)	0.00	2.13	4.98
$Ca(H_2PO_4)_2$	2.00	2.00	2.00
Choline chloride	0.20	0.20	0.20
Ethoxyquin	0.10	0.10	0.10
Calcium propionate	0.10	0.10	0.10
Vitamin premix [1]	1.40	1.40	1.40
Mineral premix [2]	1.00	1.00	1.00
Kelp powder	30.00	30.00	30.00
**Proximate analysis (dry weight %)**			
Moisture	7.32	7.48	7.52
Crude protein	30.15	30.22	30.19
Crude lipid	1.57	3.82	6.72
Ash	15.25	15.28	15.42

[1] Vitamin premix (each/kg diet): thiamine, 120.0 mg; riboflavin, 100.0 mg ; folic acid, 30.0 mg; nicotinic acid, 800.0 mg; Vitamin B6, 40mg; calcium pantothenate, 200.0 mg; inositol, 4000.0 mg; biotin, 12.0 mg; Vitamin C, 4000.0 mg; Vitamin B12, 0.18mg; Vitamin A, 100,000 IU; Vitamin D, 2000 IU; Vitamin E, 450 mg; and Vitamin K3, 80 mg. [2] Mineral premix (each/kg diet): NaCl, 0.4 g; $MgSO_4 \cdot 7H_2O$, 6.0 g; $NaH_2PO_4 \cdot 2H_2O$, 10.0 g; KH_2PO_4, 12.8 g; Fe-citrate, 1.0 g; $ZnSO_4 \cdot 7H_2O$, 141.2 mg; $CoCl_2 \cdot 6H_2O$, 0.4mg; KIO_3, 1.2 mg; $MnSO_4 \cdot H_2O$, 64.8 mg; $CuSO_4 \cdot 5H_2O$, 12.4 mg; $Na_2SeO_3 \cdot 5H_2O$, 0.61 mg.

ATTACATCGCAGATTGGAGTGTCAGTGACTGTTGGCAGTTCCATTATACGAGCC-
TATGGATGTTTAACTTTGTCTGAAGTGTGGAATATGCGGGCTGAAGACGAATAC-
CGTGGTGTGTTCTAGATATTAAGTGTTACGTTCATTTGCAAAACGCAAG ATG GAC GCT GCA GAC ATA GAT CCT AAT TTC GAG CCT CAA ACT
M D A A D I D P N F E P Q T
AGG GCA AGA TCT AAC ACC TGG CCT CTT CGA CCT AAT CGG GAG ATT CTT GAT GCC CAG AAT
R A R S N T W P L R P N R E I L D A Q N
AGC CCC GTA TCA GAG GAT TCT TCC ACT GAA AAT CAC AAG GAA AAA GAT CCA CTC GGA TTA
S P V S E D S S T E N H K E K D P L G L
AAC ACT AAA AAG AGT GGG TCA AGA CGA AAT GCG TGG GGA AAC TTA TCA TAT GCG GAT TTG
N T K K S G S R R N A W G N L S Y A D L
ATA ACG AAA GCT ATC CAA AGT TCA CCT GAA AAG CGT TTG ACT CTA TCT CAA ATA TAC GAC
I T K A I Q S S P E K R L T L S Q I Y D
TGG ATG GTA CAG AAT GTT CCT TAT TTC AAA GAT AAA GGC GAC AGT ACG AGC TCA GCT GGT
W M V Q N V P Y F K D K G D S T S S A G
TGG AAG AAT TCA ATT CGG CAC AAC TTG TCC TTG CAC AGT CGC TTC ATG CGT ATT CAG AAT
W K N S I R H N L S L H S R F M R I Q N
GAA GGA ACA GGC AAG AGT TCT TGG TGG GTT ATC AAC CCA GAT GCC AAG CCA GGG AAG ACC
E G T G K S S W W V I N P D A K P G K T
CCC AGA CGG AGG GCA GGC AGC ATG GAA ACC AAA AAC TAT GAA AAG AAG CGT GGT CGT GTT
P R R R A G S M E T K N Y E K K R G R V
AAG AAG AAA GTG GAA GCA TTA AGA GCT GCT TTA GAG GCT GGT GGA AGT CCA TCA TCA GCT
K K K V E A L R A A L E A G G S P S S A

TCA GAG GAC TTT TTA GAT ACA ACC TTT GGC TTT GAC TTT CGA CCA CGA GCT AGT TCT AAT
S E D F L D T T F G F D F R P R A S S N
GCT AGT AGC TGT GGC CGG CTT TCT CCC ATC CAA GCT GCT GTA GAA CCA GAT TTA CAT GAT
A S S C G R L S P I Q A A V E P D L H D
AAC CAG GTA CCC CCA ATG TCT CCT ATA CCA TGG GGA CCT GAA GTG GAT TCA GGG AAT TTC
N Q V P P M S P I P W G P E V D S G N F
TGT TAT AGT TCT GAT GGA TTT ACA GAT CAG TTA GTG GAC ACT CTA GTA GAC AGT ATG AAA
C Y S S D G F T D Q L V D T L V D S M K
CTA GCA GAA CCA AAT AAC ATA GGA TTG ACA GGT TCT GAA AGT TTA GAT TTG GTA GGG AAT
L A E P N N I G L T G S E S L D L V G N
GGG TTA CAA GAA CAA TTC ATG CAG AAT GGT TCT AAC AAT GAT TTG ACT ATG TCT GAT TTC
G L Q E Q F M Q N G S N N D L T M S D F
AAT CAA AAT TCA AAT TAT AGT CAA AAT GGA AGT TTC AGT GGT CAG TTT GCT AAC CTC CCA
N Q N S N Y S Q N G S F S G Q F A N L P
GCC CCA CCA CCT TAC CCT GAG TCG TTG CAA CGC AAC ACT CAG TCA CCT GCC CAG CAG TCT
A P P P Y P E S L Q R N T Q S P A Q Q S
GTG CCT CAG ACA GTG ATG AGC AAC CTA GAG CGT GTA GCC ATC TCT AAC AGT CGT TTC AAC
V P Q T V M S N L E R V A I S N S R F N
AAC TTG GGT TTG CGA CAG AAT CTG TTC GGG CAG CAA GAC TTG TAC CAA CAA GAG TCT AGC
N L G L R Q N L F G Q Q D L Y Q Q E S S
ATG AGC CAG GAC TTG GCA GCC CAA CTA AAC ACA ACC AAT CAG CTG ACT ATA AAC ACA CAG
M S Q D L A A Q L N T T N Q L T I N T Q
CCT CAG ATG ACA TCA CCT TCT CGA TCT CCA CAA CAG GCC AGC CCA ATT GGG TAC GGG AAT
P Q M T S P S R S P Q Q A S P I G Y G N
GGG TTC AGT CCA AGT GTT TCC CCA CAG CCC CAC CCA GGT CAA CCG CGC CAG CTC TCC CCA
G F S P S V S P Q P H P G Q P R Q L S P
CAG CCA CGG CAG ATG TCT CCT CAG CAG GCT CCA GGC TCC CCA GCA TCT GGC TCA GCA ACT
Q P R Q M S P Q Q A P G S P A S G S A T
GTC CCG CCC CGC ACG GTG CTG CAG CAG CAG CTC CAA TCT TCC AAT GAC TCC ATC CTG CGA
V P P R T V L Q Q Q L Q S S N D S I L R
GCA GCA CTC ACC CAA GGA GGG GCC GCC ATG AGT TAC ACC CTG CCC ACC ACG ACT GCC AGT
A A L T Q G G A A M S Y T L P T T T A S
CAC TCA CAG TTC TCA CAG ATG AAC ATG CCT TAC TCC CGT GCC CAA CTG AAT GGC CCT GTT
H S Q F S Q M N M P Y S R A Q L N G P V
TCA ACC ACA TGT AAC AAT ATG GAC AGT CAA GAC TCC AAC ATG GAT GAG GTC CTT GCA ACT
S T T C N N M D S Q D S N M D E V L A T
CAA CTT GGC CAG CAG CTT AAC AAC CAG AGA AGC CAA ACA CCT TTA CAG TCG GGA AGC TTT
Q L G Q Q L N N Q R S Q T P L Q S G S F
CCA AAC ACA GGC TCT GGC AAC ATG AAC AGT GGA CCC GGT GAG GGA GAT GTA CCT ATT GAT
P N T G S G N M N S G P G E G D V P I D
ATA GAT ATA GAA AAC TTG ATA TTT GGA TTG GAC TGT GAC CTT GAT CAG ATC ATT AAG CAA
I D I E N L I F G L D C D L D Q I I K Q
GAG TTG TCT CTA GAA GGC AAA TTA GAC TTT AAC TTT GAT GGG AGT GCC AAT GTT CCT CCA
E L S L E G K L D F N F D G S A N V P P
GCT ACC AAC AAG AAT ACT GTT TGA TGATACTCATGGTGGCGTTATTTAGTGCTGTACTTATGTGAT
A T N K N T V *

GTGATGTATTGTGATGTGATTATGTATGAGGAACCATGGCTAATGAGAAACCAGTATGGAT-
TCTGTGGCACAAGAATGGTGAGGCAATGGTAGCTATATAGGGCAGCATGCCTGTTTGCTAG-
CAGGCGAGCAGAGACATTTGAAGATCAGGGAGGTTGTCGGTCA

Figure S1. Nucleotide and deduced amino acid sequences of FoxO in abalone. The forkhead (FH) domain (64–153aa) is marked in yellow and a transactivation (FoxO-TAD) domain (590–622aa) is underlined.
